# Comparing preservation substrates under field conditions for efficient DNA recovery in bone

**DOI:** 10.1007/s00414-022-02923-w

**Published:** 2022-12-10

**Authors:** Jorge Adrián Ramírez de Arellano Sánchez, José Miguel Moreno Ortiz, Andres López Quintero, Heidi Pfeiffer, Marielle Vennemann, Hannah Bauer

**Affiliations:** 1 Department of Molecular Biology and Genomics, Universidad de Guadalajara, Guadalajara, México; 2 Institute of Legal Medicine, University of Münster, Münster, Germany

**Keywords:** Missing person identification, Bones, DNA extraction, Solid NaCl

## Abstract

Often bones are the only biological material left for the identification of human remains. As situations may occur where samples need to be stored for an extended period without access to cooling, appropriate storage of the bone samples is necessary for maintaining the integrity of DNA for profiling. To simulate DNA preservation under field conditions, pig rib bones were used to evaluate the effects of bone cleaning, buffer composition, storage temperature, and time on DNA recovery from bone samples. Bones were stored in three different buffers: TENT, solid sodium chloride, and ethanol-EDTA, at 20 °C and 35 °C for 10, 20, and 30 days. Bones were subsequently dried and ground to powder. DNA was extracted and quantified. Results show that temperature and storage time have no significant influence on DNA yield. DNA recovery from bones stored in solid sodium chloride or ethanol-EDTA was significantly higher compared to bones stored in TENT, and grinding of bones was facilitated by the extent of dehydration in solid sodium chloride and ethanol-EDTA compared to TENT. Overall, solid sodium chloride was found to be superior over ethanol-EDTA; when it comes to transportation, dry material such as salt eliminates the risk of leaking; it is non-toxic and in contrast to ethanol not classified as dangerous goods. Based on this study’s results, we recommend NaCl as a storage substrate for forensic samples in cases where no cooling/freezing conditions are available.

## Introduction

New clandestine burial pits, or mass graves, are constantly being discovered worldwide. As defined by the United Nations, a mass grave is a grave containing three or more human corpses, usually victims of execution. In Iraq, over 200 mass graves containing up to 12,000 victims have been opened up; in Bosnia, 94 graves and 337 surface areas have been recovered containing 17,000 sets of human remains from which almost half have been identified so far [1]. In Mexico, almost 2000 clandestine graves have been discovered and opened in 24 of the 32 states, with almost 3000 bodies, over 300 craniums and over 200 bones, about 800 bone remnants, and thousands of other remains recovered. Of those bodies, over half have been identified. Additionally, there are 37,000 unidentified remains being held in forensic services, several thousand graves yet to be opened, and more than 70,000 people are officially still missing [2, 3]. Over the last decade, there has been a steady increase in the number and types of both professional and lay people assisting in tracking down sites of clandestine graves as well as communicating their social, political, and cultural context [4]. To provide relatives with certainty by identifying the unknown deceased, law enforcement spends substantial resources to identify human remains, and for assisting in the investigation, the expertise of forensic scientists from particularly countries in Western Europe or the USA is generally sought. Not because of the lack of knowledge of the countries concerned but more so because of political reasons [5]. After the recovery of human remains from clandestine graves, samples are taken for further investigation, such as soft tissue or bone for DNA profiling. Genetic typing of short tandem repeats (STR) is currently the gold standard for DNA profiling for human identification. To obtain a STR profile suited for comparison to ante mortem data, such as reference profiles from living relatives, high quantity and quality DNA is needed [6]. However, limited facilities, unique environmental conditions such as humidity or temperature, or no access to cooling might affect the DNA quality, resulting in incomplete STR profiles and hindering correct identification [7]. Hence, maintaining the integrity of DNA for successful STR typing of human remains may be reliant on the immediate preservation of the sample. Several preserving methods have been described when in-field to avoid DNA degradation [8, 9], however, not all show the same protection level. Therefore, it is essential to evaluate their performances under different conditions, reflecting real situations during sample transportation. As it is common to obtain samples and have them analyzed in a different place, in some cases even in a different country or continent, various substrates were evaluated in this study for their success in maintaining DNA integrity over time and under different storage conditions during transport. From previous literature, three substrates with the most promising results regarding DNA recovery were evaluated: TENT buffer [8], solid NaCl [10], and ethanol-EDTA [10] (slightly modified). Since pigs resemble humans anatomically and physiologically [11], an animal model using pork spare rib bone was initially used in this study and subsequently applied to human bone. Samples were cleaned manually of soft tissue either roughly or completely to evaluate potential influence of flesh remains on DNA degradation, and then stored in the different substrates for up to 30 days at room temperature (20 °C) and 35 °C.

## Materials and methods

All of the following analyses were performed at the Institute of Legal Medicine, Münster, Germany.

### Sample set and preparation

After removing the flesh either completely or roughly from the pork spare rib bones, they were cut into samples of approximately 2 g using an electric rotary tool.

### Storage substrates and conditions

A total of 108 bone samples were processed in this study plus one positive control (fresh clean bone extracted after freezing without storage time), and the following conditions were applied: three storage substrates were evaluated for their suitability to preserve DNA from bone samples: TENT buffer (Tris 10 mM, EDTA 10 mM, NaCl 1 M, Tween20 2%, pH 8), solid sodium chloride (NaCl; molecular grade), and 99% ethanol(EtOH)-EDTA (10 mM, pH 9). To triplicates of 50-mL FalconTM tubes filled with either 40 mL TENT, 40 mL EtOH-EDTA, or 46 g NaCl (1:24 salt-bone-ratio), one bone sample was added. For 10, 20, or 30 days, half of the tubes were stored at room temperature (RT; 20 °C) and half at 35 °C. At each time point, bone samples were rinsed with tap water followed by HPLC-grade water to remove the storage substrate and immediately stored at − 80 °C for 2 h. The frozen bone samples were ground to powder using the TissueLyser II with a stainless-steel grinding jar set (Qiagen, Germany) at 30 Hz/s for 60 s. Every processing batch of 36 bones consisted of three replicates for each condition.

### DNA analysis

DNA was extracted using The AutoMate ExpressTM Forensic DNA Extraction System with the PrepFilerTM Express BTA kit (Thermo Fisher Scientific, Darmstadt, Germany) to a 50-μL elution volume according to manufacturer’s instructions. Fluorometric DNA quantitation was performed using the Quantus™ Fluorometer with the QuantiFluor® dsDNA System (Promega, Walldorf, Germany) according to manufacturer’s instructions.

Additionally, triplicates of a human clavicle were processed according to the pig bone samples and stored for 10, 20, and 30 days and 35 °C in the substrate shown to result in the highest DNA recovery in pig bone. To check for STR profile quality, DNA from the positive control as well as the stored samples was amplified using the PowerPlex® ESX 17 (Promega, Mannheim, Germany) and the GlobalFiler™ PCR kits (Thermo Fisher Scientific, Darmstadt, Germany) according to manufacturer’s recommendations. The samples were analyzed using the 3130 Genetic Analyzer with the GeneMapper® ID software by Thermo Fisher Scientific.

### Statistical analysis

Statistically significant differences between storage conditions were calculated using the Mann–Whitney *U* test. A value of *p* < 0.01 was considered statistically significant; a value of *p* < 0.001 was considered highly statistically significant. The calculations and descriptive statistics were carried out using SPSS version 28.

## Results

**Fig. 1:**
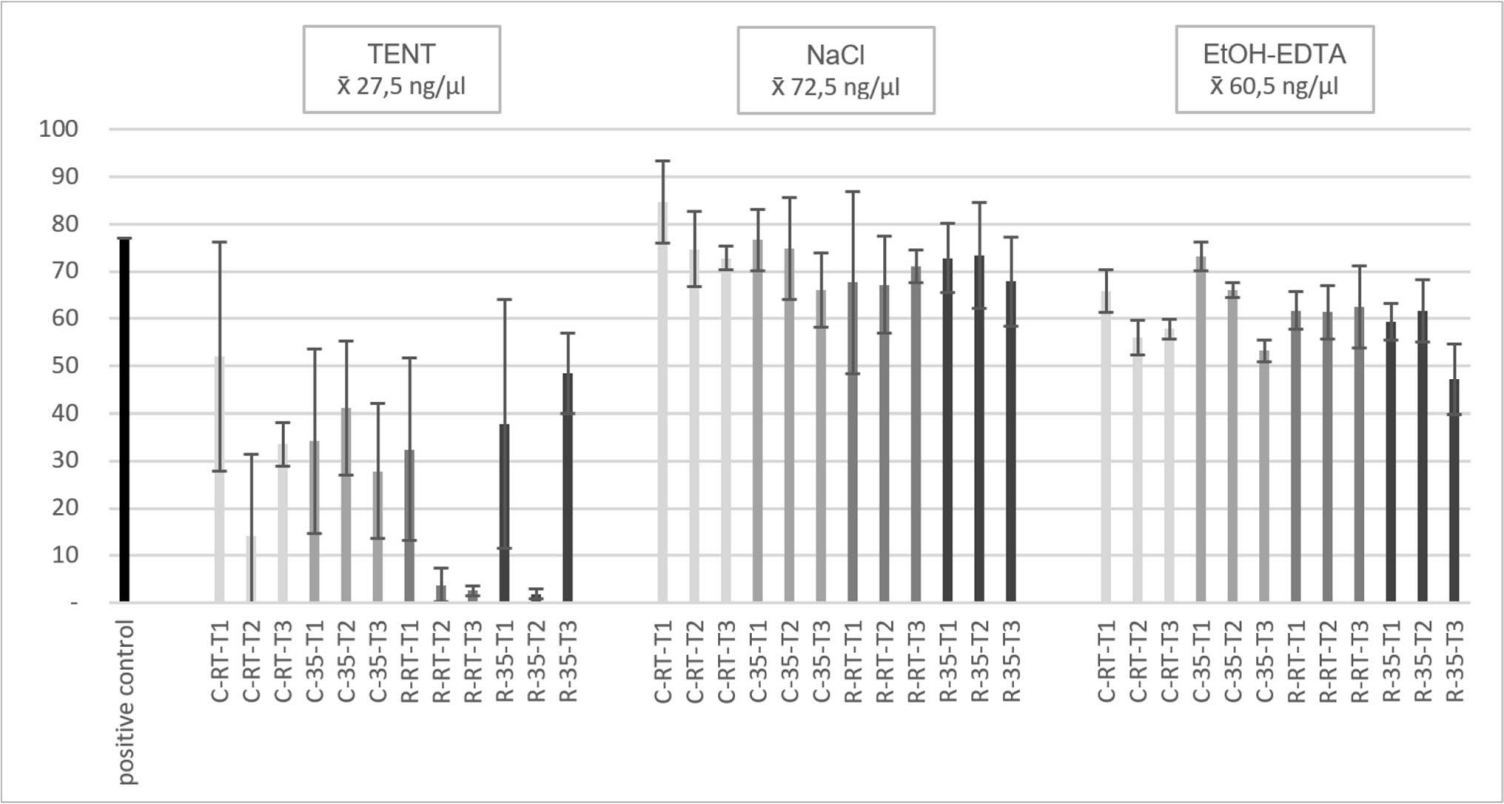
DNA concentration (ng/μl) of bone stored in TENT, NaCl, and EtOH-EDTA after 10 (T1), 20 (T2), and 30 (T3) days of storage with the mean standard variation. Samples were cleaned completely (C) or roughly (R) before being stored at RT or 35 °C. Each bar represents the mean of the triplicates

The capacity of the three storage substrates to preserve DNA from bone was tested with 108 samples. It was possible to extract DNA from all of the samples. The positive control contained 77.0 ng/μL of DNA. It was observed that cleaning of the bones as well as storage temperature and time had an impact on DNA recovery; on average, complete cleaning of the bones elevated DNA recovery compared to rough cleaning (x̄ 33.8 ng/μL vs. x̄ 21.2 ng/μL). Mean DNA recovery was higher at 35 °C storage than at RT (x̄ 31.9 ng/μL vs. x̄ 23.1 ng/μL). However, the differences in DNA recovery were not statistically relevant (*p* = 0.219 and *p* = 0.919, respectively). With a mean DNA concentration of x̄ 59.9 ng/μL, DNA yield was highest after 10 days compared to x̄ 49.7 ng/μL and x̄ 50.9 ng/μL after 20 and 30 days, respectively. However, the loss of DNA over time was not statistically relevant (10 vs. 20 days; *p* = 0.546, 10 vs. 30 days; *p* = 0.976, 20 vs. 30 days; *p* = 0.358). Buffer composition showed to have the highest influence on DNA yield after storage (Fig. 1): with an average DNA concentration of x̄ 27.5 ng/μL, samples stored in TENT buffer resulted in the lowest DNA recovery. Compared to the positive control, DNA recovery from samples stored in TENT ranged from 2.5 to 68%. DNA loss in TENT was statistical highly relevant compared to storage in EtOH-EDTA and NaCl (*p* < 0.001, each). With an average DNA concentration of x̄ 60.5 ng/μL, samples stored in EtOH-EDTA buffer resulted in about twice the DNA amount as samples stored in TENT buffer. Compared to the positive control, DNA recovery from samples stored in TENT ranged from 61 to 95%. DNA loss in EtOH-EDTA was statistical highly relevant compared to storage in NaCl (*p* < 0.001). With an average DNA concentration of x̄ 72.5 ng/μL, samples stored in solid NaCl yielded the highest DNA recovery of all three substrates. Compared to the positive control, DNA recovery from samples stored in solid NaCl ranged from 86 to 110%. When focusing on the most successful substrate regarding DNA recovery, solid NaCl and RT were superior over storage at 35 °C (x̄ 73 ng/μL vs. x̄ 50.9 ng/μL). The loss in DNA recovery with elevated temperatures was, however, not statistically relevant (*p* = 0.552). Complete cleaning of the bone was also beneficial (x̄ 74.9 ng/μL vs. x̄ 70 ng/μL), while again, not statistically relevant (*p* = 0.406). Shorter storage time was advantageous (x̄ 70.2 ng/μL at 10 days, x̄ 66.9 ng/μL at 20 days, and x̄ 62.3 ng/μL at 30 days), and the loss in DNA was statistically relevant when 10 and 30 days were compared (*p* = 0.01).

As solid NaCl showed to be the most promising substrate for DNA preservation in pig bone, it was tested whether this method was applicable to human bone. DNA recovery from the human samples compared to the human positive control ranged between 36 and 86%. After 10 days of storage, 75.9 ng/μL of DNA was recovered; after 20 days, the DNA yield was 61.0 ng/μL and 79.2 ng/μL after 30 days. STR profiles from the stored human clavicle generated with both kits showed to be of high quality for up to 30 days of storage: all of the expected alleles were detected with well-balanced peak heights across all loci, and no signs of DNA degradation were observed.

## Discussion

Clearly, the variation in DNA recovery in pig bone was mostly dependent on the storage substrate rather than cleaning or temperature conditions. NaCl and EtOH-EDTA showed to be superior over TENT in terms of DNA recovery after an extended period of storage time, which supports findings of previous studies [9, 10, 12, 13]; Allen-Hall et al. concluded that the EtOH-EDTA buffer is suitable to preserve tissue for up to 28 days at 35 degrees, and reported excellent DNA quality of the DNA extracted [10]. Brining with salt has been used for meat preservation for centuries and its high effectivity in preserving biological samples has been reported in numerous publications; Allen-Hall et al. tested the effect of solid NaCl on the preservation of muscle tissues and found it to be an excellent substrate to preserve DNA for up to 28 days [10]. Caputo et al. evaluated the preservation of DNA in samples stored for up to 365 days, concluding that solid salt is an easy, economical, and effective method to preserve biological samples that require transportation [13]. Even though TENT has been reported to help maintain DNA integrity in bone samples at room temperature [8], these findings were not confirmed in our study, with TENT yielding the lowest DNA recovery. It was reported that a TENT buffer has also been used in muscle tissues with different temperature and humidity conditions, where it led to high quantity of DNA but with rather poor quality [10], resulting non-optimal STR typing results [7]. However, although the results of the quantification of DNA from pig bone samples indicate the presence of sufficient DNA for STR analysis, we obtained no information regarding DNA quality. While for human DNA highly sensitive quantification kits are commercially available containing human-specific primers and probes and reveal information on DNA degradation and the presence of inhibitors [14], similar systems are currently not easily available for pig DNA. Consequently, in the pig samples, DNA damage may have occurred, which is not detectable by QuantiFluor analysis, and the presence of microbial DNA may be further misleading when interpreting DNA quantification results [15]. As successful STR profiling not only depends on quantity but also highly on the quality of DNA extracted from biological samples and the two might not be necessarily linked [16], a human clavicle was analyzed by example in this study as it gave us the possibility to not only quantify the samples but generate STR profiles as a quality check. Results showed that after storing bone samples even for an extended period at elevated temperatures, full STR profiles of high quality were obtained, and thus, the proof of principle obtained from pig bone is indeed applicable to human bone.

## Conclusion

While EtOH-EDTA and NaCl were comparably successful in terms of DNA preservation, we found NaCl to be the preserving method of choice: not only was it highly effective at preserving biological samples, it also requires minimal preparation, it is easily accessible, comparably cheap, and no special equipment or training is needed. When it comes to transportation, dry material such as salt also eliminates the risk of leaking; it is non-toxic and in contrast to EtOH not classified as dangerous goods. Based on this study’s results, we recommend NaCl as a storage substrate for forensic samples in cases where no cooling/freezing conditions are available.

## Data Availability

This is the final statement in the conclusion and refers to the entire article, which provides all data.
